# Pharmacogenomics: Activating Cancer Drug Discovery

**DOI:** 10.1289/ehp.112-1277127

**Published:** 2004-08

**Authors:** M. Nathaniel Mead

Normal cells that transform into cancer cells undergo various metabolic changes, including shifts in activities of enzymes that mediate macromolecule synthesis and growth-signaling pathways. Proteomics technology now provides an elegant way to identify the enzymes that are active in processes linked with tumor progression. As demonstrated by a recent study conducted at The Burnham Institute’s Cancer Center in La Jolla, California, this approach is beginning to unveil some novel high-efficacy targets for cancer control and treatment.

In work published 15 March 2004 in *Cancer Research*, the Burnham team used a novel proteomics screen based on probes that bind to the active site of the enzyme target. By competing with such probes for the active site, one can simultaneously identify protein targets and screen for their inhibitors. Activity-based proteomics screening is fast emerging as “the wave of the future,” says coauthor Steven J. Kridel, a postdoctoral fellow at the time of the research and now an assistant professor of cancer biology at Wake Forest University of Winston-Salem, North Carolina—it enables the generation of hypotheses that can lead to meaningful clinical applications. The chemical strategy for activity-based proteomics was pioneered in the laboratories of cell biologist Ben Cravatt of The Scripps Research Institute and pathologist Matthew Bogyo of Stanford University. Kridel and colleague Jeffrey Smith, associate scientific director for technology at The Burnham Institute, are among the first to use the approach to identify a therapeutic lead.

The activity-based strategy may mark a major improvement over the usual proteomics approaches, which are based on the relative abundance of a particular protein target. “Measuring the abundance of a protein only provides a static picture of a potential target enzyme,” says Kridel. “There are several levels of regulation between protein abundance and protein activity. With activity-based proteomics, you also can tell whether there is a specific physiologic state that turns off the enzyme’s activity and whether an inhibitor of that particular enzyme exists.”

Kridel and Smith applied the activity-based strategy to identify proteins that exhibit different activities in cancer cells as compared to normal cells. They screened a group of enzymes known as serine hydrolases by measuring the activity levels of these enzymes in normal prostate epithelial cells and in three standard prostate cancer cell lines. They found that serine hydrolase expression was generally similar among all cell lines, with two key exceptions: one of the hydrolases was active in normal prostate cells but virtually inactive in all the tumor cells, while another was expressed in all of the tumor lines but absent in the normal cells. The latter enzyme was shown to be fatty acid synthase (FAS), which had earlier been strongly linked to tumor progression, making it an attractive therapeutic target.

Having identified their molecular target of choice, the investigators then screened possible inhibitor drugs, hoping to find unforeseen side benefits in drugs already approved for human use. “Our goal from the outset was to find an anticancer drug that might not have been considered before,” says Kridel. “We wanted a drug that inhibits a protein that is only expressed in cancer cells, not in normal cells, in part because we believed this would minimize toxic side effects.” Among the many agents reviewed was the anti-obesity drug orlistat (trade name Xenical). Kridel says orlistat had not previously been shown to inhibit FAS, and FAS inhibition is not believed to be relevant to orlistat’s mode of action in weight loss.

In cell culture studies, the Burnham team found that orlistat inhibited proliferation and induced apoptosis in at least two lines of prostate cancer cells. The antiproliferative effects were reversed by the addition of palmitate, the precursor for the majority of nonessential fatty acids, which cancer cells use primarily for energy and growth. This strongly implicated FAS inhibition, as FAS is the only eukaryotic enzyme capable of synthesizing palmitate. In rodent experiments, orlistat blocked tumor growth significantly, and the animals showed no outward signs of toxicity or adverse changes in blood chemistry.

By revealing some of the unanticipated effects of a drug, activity-based proteomics could markedly reduce the cost of drug development. “Orlistat just happens to be an approved drug with relatively minor toxicity that could be utilized quickly once its effectiveness in human prostate cancer is validated,” says Massimo Loda, an associate professor of pathology at Harvard Medical School and the Dana Farber Cancer Institute in Boston, Massachusetts. “The implications of this study are dual: this activity-based proteomics approach can now be applied to the screening of diverse families of enzymes that sustain tumor survival, and it may reveal unsuspected activity of known drugs utilized in diseases other than cancer.”

Such research may eventually pave the way for construction of a proteomics profile of susceptibility to cancer progression. “If a man presents with prostate cancer and has a biopsy, it is entirely possible that the proteomics screening approach can be used to assess whether his tumor has upregulated FAS,”

Smith says. “If it does, you can then prescribe a specific treatment regimen: to reduce dietary fat and block FAS activity using orlistat. This is moving toward personalized medicine.” Smith believes a low-fat diet could reinforce orlistat’s cancer-fighting effects in humans. “We know that tumor cells have a unique requirement for fat,” he says. “If you restrict dietary fat and knock out the tumor’s ability to synthesize its own fat from carbohydrates, then the antitumor effect should be even greater.”

## Figures and Tables

**Figure:**
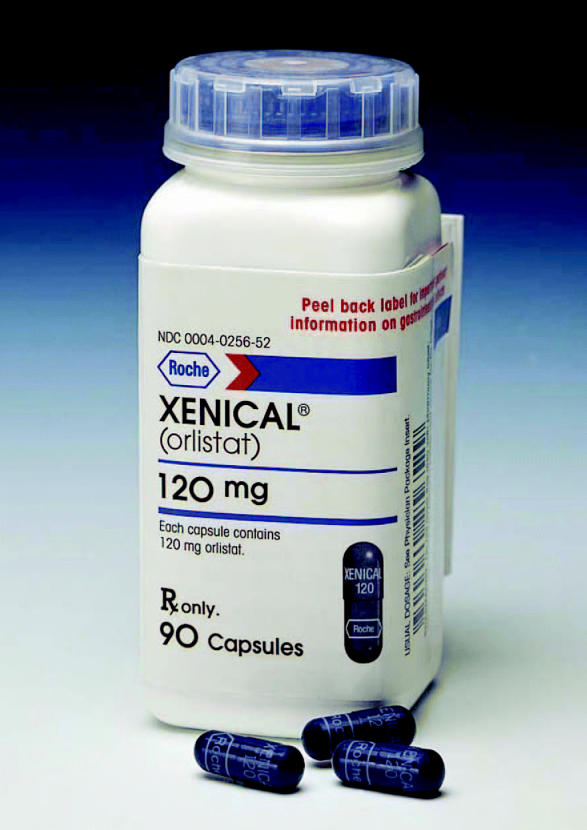
**Dual-purpose drug?** A novel activity-based proteomics screen of the weight-loss drug orlistat revealed its surprising potential as a cancer treatment.

